# Origin and Evolution of Dengue Virus Type 3 in Brazil

**DOI:** 10.1371/journal.pntd.0001784

**Published:** 2012-09-06

**Authors:** Josélio Maria Galvão de Araújo, Gonzalo Bello, Hector Romero, Rita Maria Ribeiro Nogueira

**Affiliations:** 1 Laboratory of Molecular Biology for Infectious Diseases and Cancer, Federal University of Rio Grande do Norte, Natal, Brazil; 2 Laboratorio de AIDS e Imunologia Molecular, Instituto Oswaldo Cruz – FIOCRUZ, Rio de Janeiro, Brazil; 3 Laboratorio de Organización y Evolución del Genoma, Sección Biomatemáticas, Facultad de Ciencias, Universidad de la República, Montevideo, Uruguay; 4 Laboratory of Flavivirus, Oswaldo Cruz Institute – FIOCRUZ, Rio de Janeiro, Brazil; Centers for Disease Control and Prevention, United States of America

## Abstract

The incidence of dengue fever and dengue hemorrhagic fever in Brazil experienced a significant increase since the emergence of dengue virus type-3 (DENV-3) at the early 2000s. Despite the major public health concerns, there have been very few studies of the molecular epidemiology and time-scale of this DENV lineage in Brazil. In this study, we investigated the origin and dispersion dynamics of DENV-3 genotype III in Brazil by examining a large number (*n* = 107) of E gene sequences sampled between 2001 and 2009 from diverse Brazilian regions. These Brazilian sequences were combined with 457 DENV-3 genotype III E gene sequences from 29 countries around the world. Our phylogenetic analysis reveals that there have been at least four introductions of the DENV-3 genotype III in Brazil, as signified by the presence of four phylogenetically distinct lineages. Three lineages (BR-I, BR-II, and BR-III) were probably imported from the Lesser Antilles (Caribbean), while the fourth one (BR-IV) was probably introduced from Colombia or Venezuela. While lineages BR-I and BR-II succeeded in getting established and disseminated in Brazil and other countries from the Southern Cone, lineages BR-III and BR-IV were only detected in one single individual each from the North region. The phylogeographic analysis indicates that DENV-3 lineages BR-I and BR-II were most likely introduced into Brazil through the Southeast and North regions around 1999 (95% HPD: 1998–2000) and 2001 (95% HPD: 2000–2002), respectively. These findings show that importation of DENV-3 lineages from the Caribbean islands into Brazil seems to be relatively frequent. Our study further suggests that the North and Southeast Brazilian regions were the most important hubs of introduction and spread of DENV-3 lineages and deserve an intense epidemiological surveillance.

## Author Summary

Dengue is a major health problem in the tropics and the incidence of dengue fever and dengue hemorrhagic fever in Brazil experienced a significant increase since the emergence of dengue virus type-3 (DENV-3). In this study, the authors reconstruct the spatio-temporal dispersion pattern of the DENV-3 lineage that circulates in Brazil and the Americas. The authors found that DENV-3 outbreaks occurring in the American continent since the mid-1990s are the result of a single introduction of genotype III. The Central American countries and Mexico were the hubs of genotype III spread in the Americas, while the Caribbean region acted as a staging post between Central America/Mexico and South America. The authors estimate that there have been at least four introductions of the DENV-3 genotype III in Brazil, although only two of them succeeded in getting established and disseminating through the country. The Lesser Antilles (Caribbean) were the main source of DENV-3 viruses that arrived into Brazil,and the North and Southeast country regions seem to be most important hubs of introduction and dissemination of DENV-3 lineages. These findings offer important information to perform more effective surveillance programs to detect introduction and dispersal of new DENV lineages in Brazil.

## Introduction

Dengue virus (DENV) is a member of the genus *Flavivirus,* family *Flaviviridae,* and one of the most important arboviral pathogens. The single-stranded positive-sense genomic RNA encodes one large open reading frame (ORF) as a polyprotein, which undergoes proteolytic processing into three structural proteins: capsid (C), membrane (M) and envelope (E); and seven non-structural proteins: NS1, NS2A, NS2B, NS3, NS4A, NS4B and NS5. DENV is transmitted to humans through the bites of infected *Aedes* mosquitoes, principally *A. Aegypti*, which is widely distributed around the tropical and subtropical regions of the world [1]. Infection with DENV causes a wide spectrum of disease manifestations, ranging from unapparent infection to severe and potentially fatal disease [2].

There are four distinct antigenic groups or serotypes of DENV (DENV-1 to DENV-4) that are causing human pandemics. A number of phylogenetically distinct lineages, termed genotypes, have been also identified within each serotype, which may differs in both geographical distribution and viral virulence/transmissibility [3], [4], [5]. Among them, the genotype III of DENV-3 has been frequently associated with severe dengue outbreaks in Asia, Africa and Latin America [6], [7], [8], [9], [10], [11], [12], [13], [14], [15], [16], [17], [18]. DENV-3 genotype III probably emerged in the Indian sub-continent around the middle 1970s and subsequently spread to other countries from Asian, Africa and the Americas [6], [19]. This genotype was first detected in the Americas during dengue fever/dengue hemorrhagic fever (DF/DHF) outbreaks in Nicaragua and Panama, in 1994 [20], [21]. In the following years the virus spread through the region using several independent routes from Central America and Mexico to the Caribbean and South America [18], [19], [22].

In Brazil, millions of dengue infections have been detected all over the country since 1986 [23]. The first autochthonous case of DENV-3 (genotype III) was reported in December 2000 in the state of Rio de Janeiro (Southeast region), from a patient with dengue fever [24]. During the summer of 2002, the newly introduced DENV-3 serotype caused one of the largest dengue outbreaks in the state of Rio de Janeiro, infecting a susceptible population that had only experienced DENV-1 and DENV-2 epidemics. In the first half of the 2002, the state reported 288,245 dengue cases, including 1.831 DHF cases and 91 deaths; which exceed the total number of DHF cases reported in Brazil from 1986 to the time of the epidemic [25]. Subsequent outbreaks of DENV-3 continued to be documented through the 2000s in Rio de Janeiro as well as in almost all Brazilian territory, revealing the rapid spread of this new serotype in the country.

Despite the public health importance of DENV-3 genotype III in Brazil, there have been very few studies of the molecular epidemiology of this DENV genotype in the country. The first study surveyed the phylogenetic diversity of a small number (*n* = 19) of Brazilian DENV-3 genotype III E gene sequences collected up to 2004 [16]; while two more recent studies have focused on the analysis of localized dengue outbreaks occurring in the state of Sao Paulo during 2006 [26], [27]. This prompted us to perform a more comprehensive study to investigate the origin, evolution, and dispersion dynamics of DENV-3 genotype III in Brazil by examining a large number (*n* = 107) of E gene sequences sampled between 2001 and 2009 from different locations within the country.

## Materials and Methods

### Virus isolation

Virus isolates were derived from human serum specimens obtained from 19 Brazilian patients with confirmed dengue virus type 3 (DENV-3) infection from Rio de Janeiro (*n* = 12), Espirito Santo (*n* = 3) and Goias (*n* = 4) states (Table 1). The case-patients included in this study had acute febrile illness with two or more of the following clinical manifestations: headache, retrobulbar pain, myalgia, arthralgia, rash and hemorrhage. Ethical clearance was obtained with the approval resolution number CSN196/96 from the Oswaldo Cruz Foundation Ethical Committee in Research (CEP 274/05), and all subjects provided written informed consent before participation. All samples were received refrigerated and stored at −70°C until tested. The viruses were isolated by inoculation into *Aedes albopictus* C6/36 cell lines [28] and the serotype was identified by indirect immunofluorescence using type-specific monoclonal antibodies [29].

**Table 1:** DENV-3 data set.

Region	Country	*N*	Sampling dates
South America	Argentina	6	2007
	Bolivia	2	2003
	Brazil	107	2001–2009
	Colombia	59	2001–2009
	Ecuador	3	2000
	Guyana	1	2002
	Paraguay	21	2002–2006
	Peru	15	2000–2005
	Venezuela	118	2000–2008
Central America and Mexico	Honduras	1	1995
	Mexico	10	1995–2007
	Nicaragua	18	1994–2008
	Panama	1	1994
Caribbean	Anguilla	1	2001
	Cuba	3	2000–2002
	Martinique	6	1999–2001
	Puerto Rico	93	1998–2007
	Saint Lucia	2	2001
	Trinidad and Tobago	2	2002
Asia	Bhutan	19	2006
	China	5	2009
	India	2	1984/2004
	Malaysia	1	2001
	Singapore	45	2004–2007
	Sri Lanka	15	1981–2000
	Taiwan	1	2006
Middle-West	Saudi Arabia	5	1997/2004
Africa	Somalia	1	1993
South Pacific	American Samoa	1	1986

### Viral RNA extraction, amplification, and sequencing of E gene region

Viral RNA was extracted from 140 µL of cell culture supernatant by use of the QIAamp Viral RNA Mini Kit (QIAGEN, Valencia, CA), according to the manufacturer's instructions. The complete E gene (1479 bp in length) was then amplified by reverse transcription-PCR (RT-PCR) as described previously [30]. Amplicons were directly sequenced in both directions using a BigDye Terminator Cycle Sequencing Ready Reaction kit (Applied Biosystems, US), 1 µM of primers combined with 200 ng of DNA, after purification using PCR purification kit (Qiagen, US). Thermocycling conditions consisted of 30 cycles of 94°C for 1 min, 60°C for 2 min and 72°C for 3 min. After purification using Centri-Sep columns (Applied Biosystems, US) the DNA was dried at 37°C, overnight. The pellet was resuspended in 10 µl of Hi-Di Formamide (Applied Biosystems, US), heated for 2 min at 95°C and kept on ice until 10 µl was loaded on an Applied Biosystems Prism 3730 Sequencer (Applied Biosystems, US).

### Sequence dataset

The sequences generated here were combined with all DENV-3 genotype III complete E gene sequences available at the GenBank by July 2010, from which the country and year of isolation were available. One sequence from Mozambique (GenBank accession FJ882575) previously identified as inter-genotype recombinant and two sequences from Brazil (GenBank accession FJ898446 and FJ898447), from which no information about country region was available, were excluded from the analysis. We also excluded four sequences that displayed anomalously long branches in the phylogenetic analysis: one from Brazil (GenBank accession AY038605), one from Puerto Rico (GenBank accession EU529696) and two from Argentina (GenBank accession EU052792 and EU052792) (data not shown). This resulted in a final data set of 564 DENV-3 genotype III E sequences (1,479 nt long) from the Americas (*n* = 469), Asia (*n* = 88), Middle-West (*n* = 5), South Pacific (*n* = 1) and Africa (*n* = 1), covering a total of 29 countries (Table 1). Nucleotide sequences were aligned using CLUSTAL X program [31]. Alignment is available from the authors upon request.

### Phylogenetic analysis

Phylogenetic analyses were performed under the GTR+I+Γ_4_ model of nucleotide substitution, selected using the jModeltest program [32]. A Maximum Likelihood (ML) phylogenetic tree was inferred for the complete data set of 566 DENV-3 genotype III E sequences with PhyML program [33], using an online web server [34]. Heuristic tree search was performed employing the SPR branch-swapping algorithm and the reliability of the phylogenies was estimated with the approximate likelihood-ratio test (aLRT) based on a Shimodaira–Hasegawa-like procedure. A Bayesian phylogenetic tree was inferred for a subset of 202 DENV-3 sequences using MrBayes program [35]. Chains were run for 10×10^6^ generations and convergence of parameters was assessed by calculating the Effective Sample Size (ESS) using TRACER v1.5 program [36], after excluding an initial 10% for each run. All parameter estimates for each run showed ESS values >100.

### Analysis of spatio-temporal dispersion pattern

The rate of nucleotide substitution per site per year (subs./site/year), the time to the most recent common ancestor (*T*mrca) and the spatial diffusion of a given DENV-3 lineage were jointly estimated using the Bayesian Markov chain Monte Carlo (MCMC) statistical framework implemented in the BEAST v1.6.1 package [37], [38]. A matrix of geographic locations was constructed based on the place of sampling for each sequence. A full model was used in which all possible reversible exchange rates between locations were equally likely (flat prior) [39]. Where two discrete locations were grouped together, the longitude and latitude used were those of the midpoint of the line connecting them. Where more than two locations were grouped, the latitude and longitude of the centroid of the polygon defined by them were used. Analyses were carried out with a Bayesian Skyline coalescent tree prior [40], under the GTR+I+Γ_4_ model of nucleotide substitution and using a relaxed (uncorrelated Lognormal) [41] molecular clock model. The MCMC analysis was run for 10×10^7^ generations and convergence of parameters (ESS>200) was assessed with TRACERv1.5 program as described above. Uncertainty in parameter estimates was reflected in the 95% highest probability density (HPD) intervals. The programs TreeAnnotator v1.5.2 and FigTree v1.1.2 (http://tree.bio.ed.ac.uk/software/figtree/) were used to summarize the posterior tree distribution and to visualize the annotated maximum clade credibility (MCC) tree, respectively.

## Results

### Diversification of DENV-3 genotype III in the Americas

**Figure 1:**
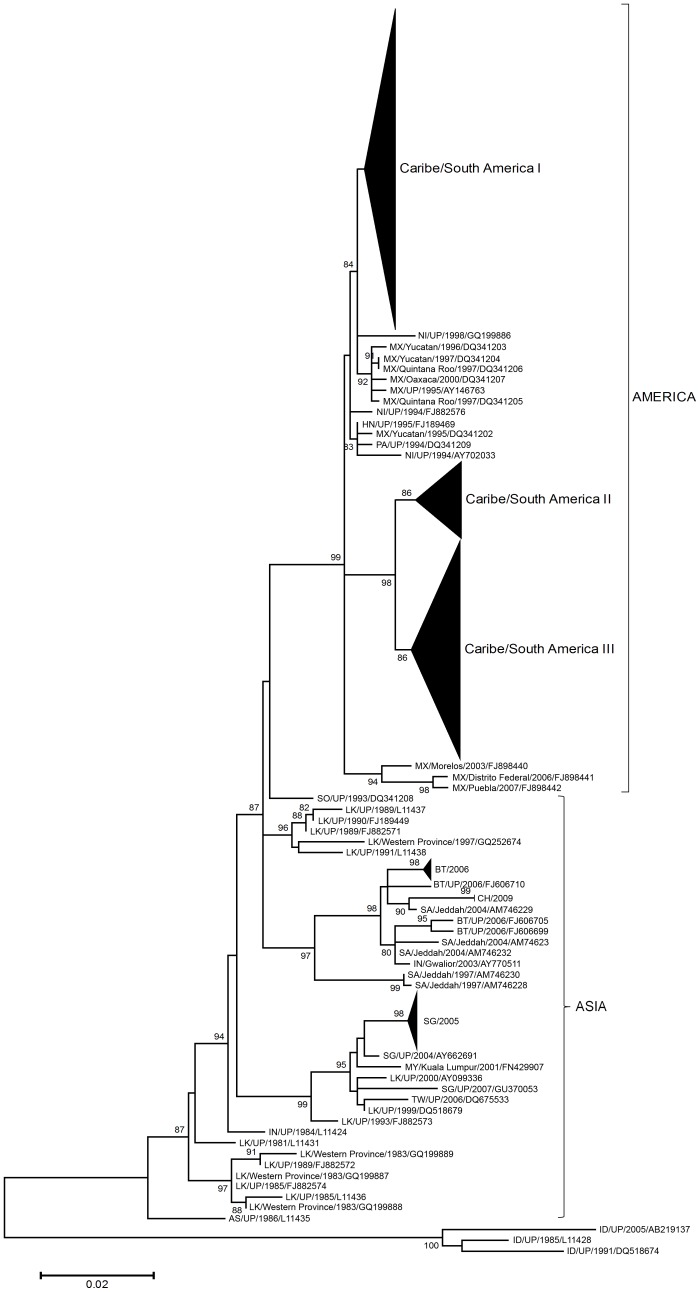
ML tree of 564 DENV-3 genotype III E gene sequences circulating globally. Brackets indicate clades comprised of sequences sampled from Asia, Africa and the Americas. Taxon labels include reference to country of isolation, year of isolation, and GenBank accession number. Country represented are American Samoa (AS), Bangladesh (BD), Bhutan (BT), China (CH), Honduras (HN), India (IN), Malaysia (MY), Mexico (MX), Nicaragua (NI), Panama (PA), Saudi Arabia (SA), Singapore (SG), Somalia (SO), Sri Lanka (LK), Taiwan (TW). For visual clarity, supported clades comprised of sequences sampled from Caribbean/South America (see Fig. 2), Bhutan and Singapore has been collapsed. Only aLTR support values >80 are shown. All horizontal branch lengths are drawn to a scale of nucleotide substitutions per site. The tree was rooted using DENV-3 genotype I strains. UP (Unidentified Place).

The phylogenetic analysis of 564 DENV-3 genotype III E gene sequences sampled world-wide revealed that all American strains segregate in a monophyletic cluster (Fig. 1), suggesting a single introduction of this genotype into the continent, consistent with previous findings [6], [19]. The only sequence of African origin included in our data set, which correspond to a virus isolated in Somalia in 1993 (GenBank accession DQ341208), branched between Asian and American strains (Fig. 1), supporting an scenario in which DENV-3 genotype III may have gone from Asia into Africa, and then into the Americas [6], [19].

**Figure 2:**
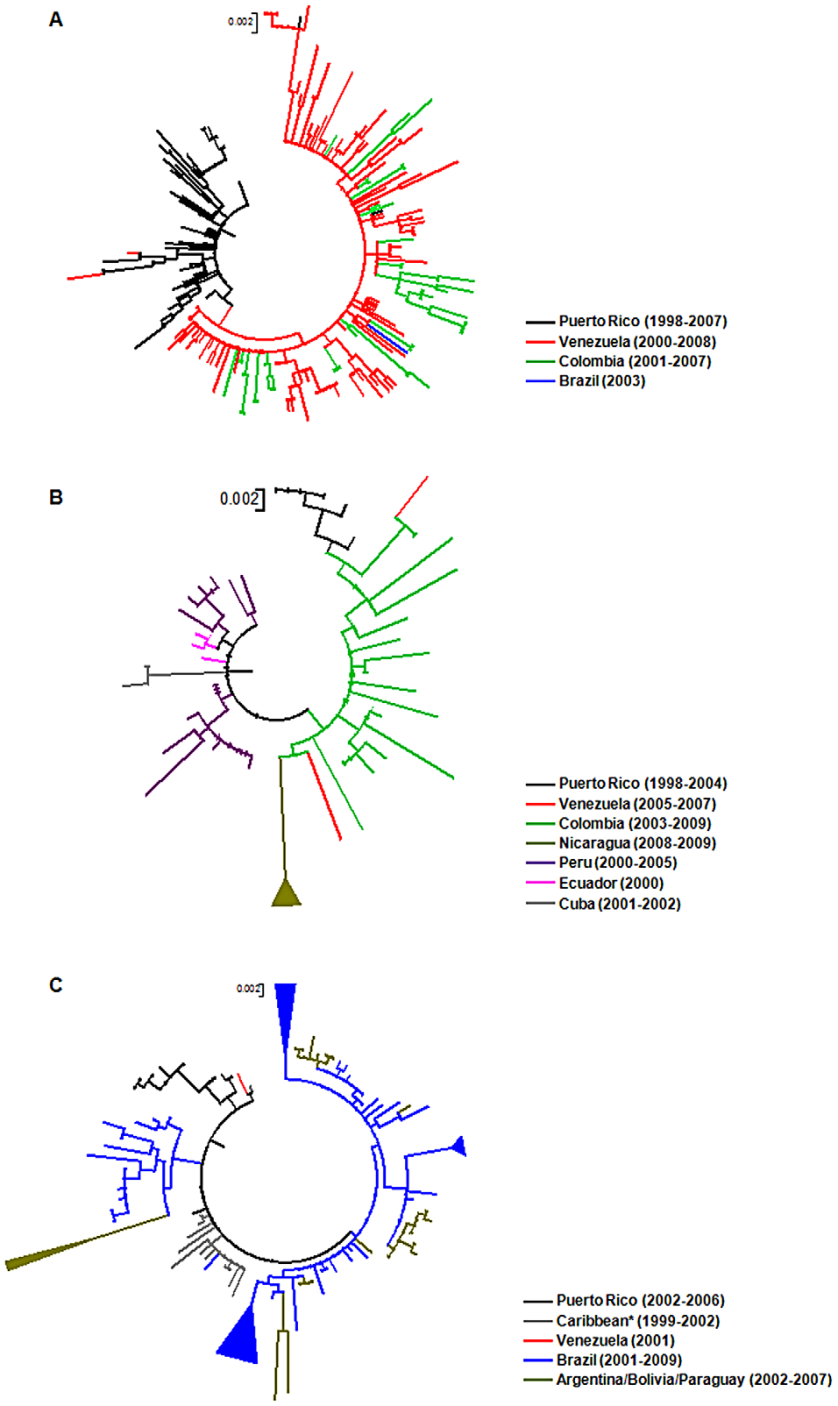
Sub-trees corresponding to the Caribbean/South America clades I (a), II (b) and III (c). The color of a tip branch represents the geographic region from where the strain originated, according to the legend given in the figure. Numbers in the legend represent the sampling date of sequences. For visual clarity, some clades composed by sequences from the same country/region are schematically represented by triangles. All horizontal branch lengths are drawn to a scale of nucleotide substitutions per site. *Caribbean: Martinique, Trinidad and Tobago, Saint Lucia and Anguilla.

Inside the DENV-3 genotype III American cluster, strains isolated in Central America (from 1994 to 1998) and Mexico (from 1995 to 2007) branched close to the root of the cluster, while sequences isolated in the Caribbean (from 1998 to 2007) and South America (from 2000 to 2009) segregate in three different monophyletic sub-clusters (Fig. 1). This pattern support the view that DENV-3 genotype III was introduced into Central America or Mexico and from there spread to the Caribbean and South America following three major routes. In the first route the virus spread to Puerto Rico, Venezuela and Colombia, producing the lineage Caribbean/South America I, which also includes one sequence isolated in Brazil (Fig. 2a). In the second route the virus disseminated into the Pacific side of South America hitting Peru, Ecuador and Colombia, and subsequently moved back to Venezuela, Cuba, Puerto Rico and Nicaragua; constituting the lineage Caribbean/South America II (Fig. 2b). In the third route the virus went to the Caribbean (Martinique, Puerto Rico, Cuba, Trinidad and Tobago, Saint Lucia and Anguilla) and from there into the Southern Cone of South America (Brazil, Argentina, Bolivia and Paraguay), originating the lineage Caribbean/South America III, which also included one sequence from Guyana and another one from Venezuela (Fig. 2c).

### Diversification of DENV-3 genotype III in Brazil

**Figure 3:**
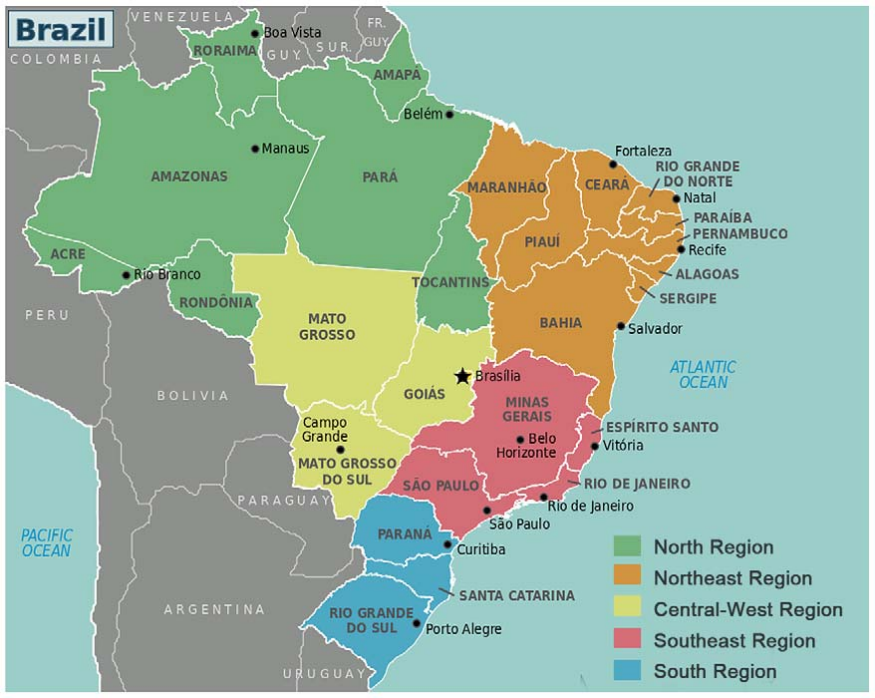
This map identifies the regions and states that make up Brazil. Legend described the color code and the number of sequences analyzed from each Brazilian region.

To analyze the diversity of DENV-3 in Brazil in more detail, we undertook a more rigorous Bayesian phylogenetic analysis of a subset of 202 DENV-3 genotype III sequences which combine all sequences sampled from Brazil (*n* = 107), along with selected non-Brazilian ‘background’ sequences (*n* = 95). DENV-3 Brazilian sequences were sampled from the Southeast (*n* = 74), North (*n* = 20), Central-West (*n* = 8) and Northeast (*n* = 5) regions (Fig. 3 and Table S1). DENV-3 non-Brazilian background sequences comprise all genotype III sequences from the lineage Caribbean/South America III (*n* = 54) and representative sequences from Asia (*n* = 10), Africa (*n* = 1), Central America and Mexico (*n* = 10), lineage Caribbean/South America I (*n* = 10), and lineage Caribbean/South America II (*n* = 10).

**Figure 4:**
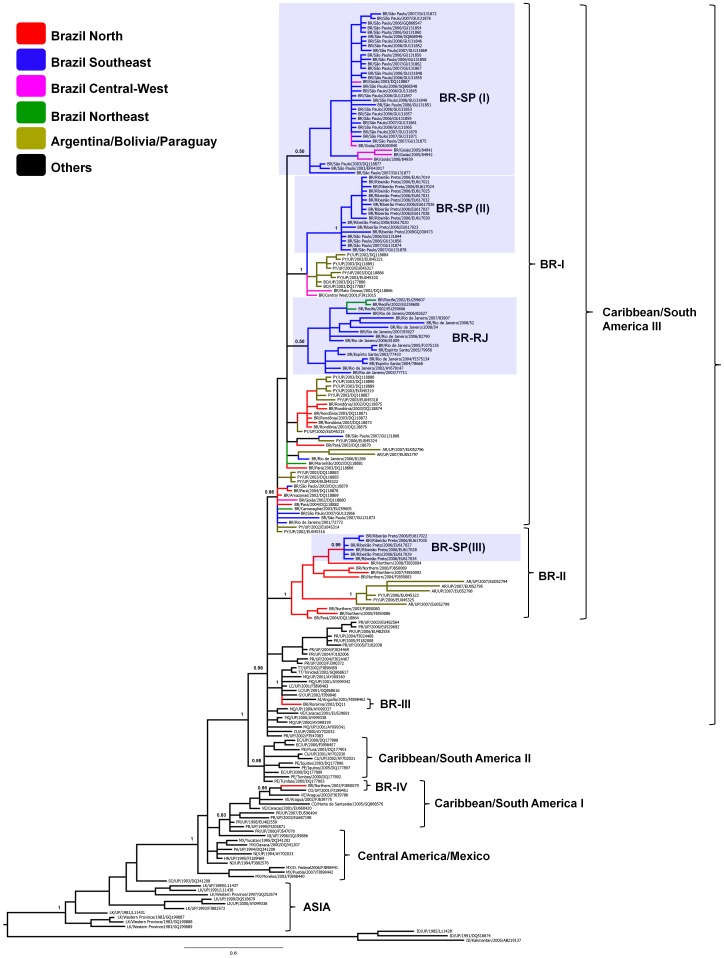
Majority-rule Bayesian consensus tree representing the global diversity of DENV-3 genotype III. The color of a tip branch represents the geographic region from where the strain originated, according to the legend given in the figure. Brackets indicate major region-specific genotype III clades and discrete Brazilian lineages (indicative of separate introductions). Broken boxes highlight subclades of Brazilian sequences circulating in Rio de Janeiro (RJ) and Sao Paulo (SP) states. Taxon labels include reference to country of isolation, year of isolation, and GenBank accession number or strain designation. Country represented are: Anguilla (AI), Argentina (AR), Bolivia (BO), Brazil (BR), Cuba (CU), Colombia (CO), Ecuador (EC), Guyana (GY), Honduras (HN), Martinique (MQ), Mexico (MX), Nicaragua (NI), Panama (PA), Peru (PE), Puerto Rico (PR), Paraguay (PY), Saint Lucia (SL), Somalia (SO), Sri Lanka (LK), Trinidad and Tobago (TT), and Venezuela (VE). PP values are shown for relevant nodes. All horizontal branch lengths are drawn to a scale of nucleotide substitutions per site. The tree was rooted using DENV-3 genotype I strains. UP (Unidentified Place).

The DENV-3 genotype III Brazilian sequences analyzed were distributed in four independent lineages, revealing at least four introduction events of this DENV-3 genotype in the country (Fig. 4). Most Brazilian sequences (*n* = 92; 86%) grouped in a well supported monophyletic clade (*PP* = 0.86) within the Caribbean/South America III cluster, called BR-I; which contains sequences sampled from all Brazilian regions from 2001 to 2009, along with sequences isolated in Argentina, Bolivia and Paraguay. A minor proportion of Brazilians sequences (*n* = 13; 12%) isolated in the North region from 2003 to 2008 and Sao Paulo state (Southeast region) in 2006, segregate in a second monophyletic clade (*PP* = 1) within the Caribbean/South America III lineage, called BR-II; which also includes sequences isolated in Paraguay and Argentina. The third Brazilian lineage is represented by a single isolate sampled in the state of Roraima (North region) in 2002 (GenBank accession DQ118865), which formed a monophyletic cluster (*PP* = 1) with sequences from several Caribbean Islands within the Caribbean/South America III clade. The fourth Brazilian lineage also correspond to a single sequence isolated in the North region at 2003 (GenBank accession FJ850079), that is closely related to Venezuelan and Colombian sequences from clade Caribbean/South America I.

Most DENV-3 Brazilian sequences included in the present study were retrieved from Sao Paulo (*N* = 58; 54%) and Rio de Janeiro (*N* = 13; 12%), which are the most populated states of the country. A closer inspection of those DENV-3 strains reveals a significant difference in the pattern of viral dissemination within these regions. While nearly all sequences isolated in Rio de Janeiro from 2002 to 2008 segregate in a single monophyletic cluster (BR-RJ), sequences sampled in Sao Paulo split in three major independent lineages: BR-SP-I (from 2003 to 2007), BR-SP-II (from 2006 to 2009) and BR-SP-III (at 2006) (Fig. 4). Furthermore, sequences sampled in Sao Paulo were closely related to Brazilian sequences isolated in the Central-West (BR-SP-I, BR-SP-II) and North (SP-III) regions; while sequences from Rio de Janeiro showed a closer relationship with sequences isolated in the states of Espirito Santo (Southeast region) and Pernambuco (Northeast region) (Fig. 4). A few DENV-3 sequences from Rio de Janeiro and Sao Paulo branched outside the major clades and possibly represent viruses that did not succeed in getting established in those regions.

### Origin and time-scale of DENV-3 clades BR-I and BR-II

In order to gain insight into the place and timing of introduction of major DENV-3 Brazilian lineages (BR-I and BR-II), we used a Bayesian MCMC phylogeographic approach that jointly estimates the substitution rate, the *T*mrca and the spatial diffusion from sampled sequences, while accommodating phylogenetic uncertainty arising from the sequence data. DENV-3 sequences from clades BR-I and BR-II were combined with Caribbean DENV-3 sequences from lineage Caribbean/South America III and with DENV-3 sequences isolated in Central America at the middle 1990s. A specific “character state” was assigned to each DENV-3 sequence based on its geographic origin, according to the following scheme: Central America (Panama, Nicaragua, and Honduras), Greater Antilles (Puerto Rico and Cuba), Lesser Antilles (Anguilla, Martinique, Saint Lucia, and Trinidad and Tobago), South America (Guyana and Venezuela), North Brazil, Southeast Brazil, Northeast Brazil, and Central-West Brazil. Analyses were performed under an equal rates model that assumes the same rate of virus movement between the eight locations.

**Figure 5:**
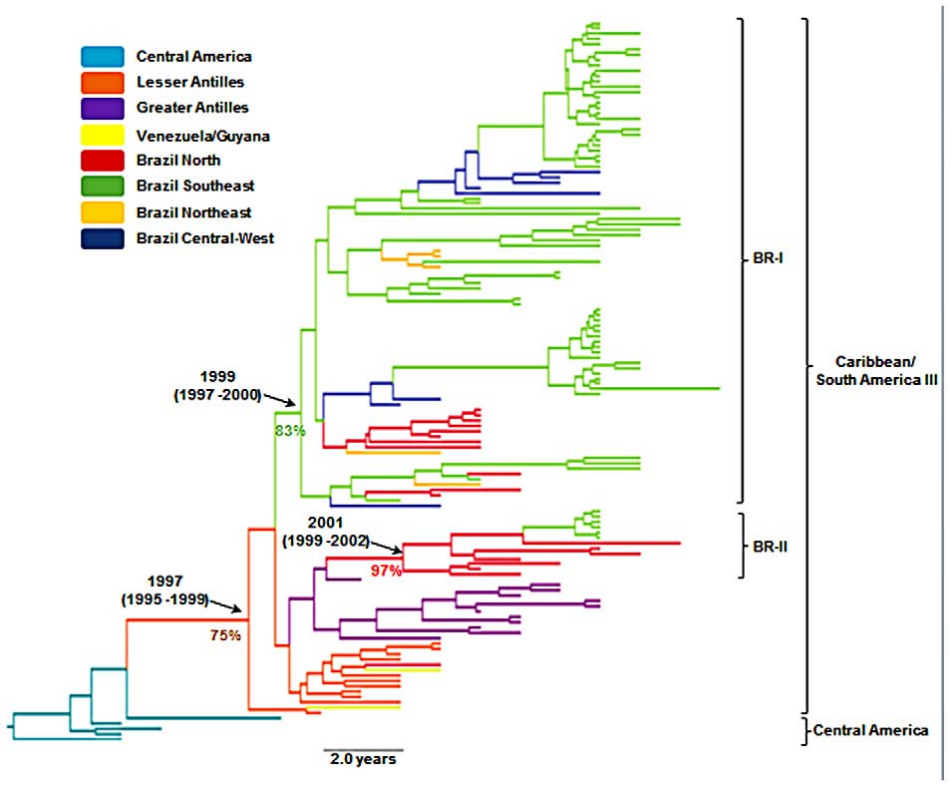
Time-scaled Bayesian Maximum Clade Credibility tree for the DENV-3 Caribbean/South America III lineage. Terminal branches of the tree are colored according to the sampled location of the taxon at the tip. Internal branches are colored according to the most probable location of their parental node. The age (with 95% HPD in parentheses) and the posterior probabilities for the geographic locations of parental nodes of the Caribbean/South America III, BR-I and BR-II lineages are shown. Branch lengths of the trees correspond to length of time (see the time scale bar). The tree is automatically rooted under the assumption of a relaxed molecular clock.

The mean evolutionary rate and *T*mrca of the DENV-3 data-set were estimated at 11.0×10^−4^ subs./site/yr (95% HPD: 8.3–13.8×10^−4^ subs./site/yr) and 1991 (95% HPD: 1988–1993), respectively; which are close to those previously reported for the DENV-3 genotype III in the Americas [19]. The spatio-temporal reconstruction suggests that the Caribbean/South America III lineage likely originated in the Lesser Antilles (*PP* = 75%) at around 1997 (95% HPD: 1995–1999), and rapidly spread to the Greater Antilles and South America (Fig. 5). The Brazilian clade BR-I was probably imported from the Caribbean islands into the Southeast region (*PP* = 83%) at around 1999 (95% HPD: 1997–2000), while the clade BR-II probably migrated from the Caribbean islands to the North Brazilian region (*PP* = 97%) at around 2001 (95% HPD: 1999–2002) (Fig. 5).

## Discussion

The genotype III has established as the major lineage of DENV-3 in the Americas. The phylogenetic analysis presented here confirms that DENV-3 outbreaks occurring in the American continent since the mid-1990s are the result of a single introduction of genotype III. This analysis also suggest that viral introduction probably occurs through Central America or Mexico, and from there the virus spread to the Caribbean and South America following three major routes, giving rise to three independent evolutionary lineages (Caribbean/South America I to III), consistent with previous findings [18], [19], [22]. According to this model, Central American countries and Mexico were the hubs of genotype III spread in the Americas, while the Caribbean region acted as a staging post between Central America/Mexico and South America. The lack of evidence of dissemination of new DENV-3 strains in the Americas reveals that despite massive human movement between continents, the establishment of new DENV-3 lineages of Asian and/or African origin in the Americas seems to be an improbable phenomenon. In the last two decades, the current lineages of DENV-3 circulating in the Americas immunized “naturally” the population, due to its wide spread in the continent. This factor may have been decisive to explain the lack of new introductions of Asian and/or African lineages.

Brazil has been heavily affected by DENV-3 since the early 2000s and our study reveals that there have been at least four separate introductions of the genotype III into the country, as signified by the presence of four phylogenetically distinct lineages. Three lineages (BR-I, BR-II, and BR-III) belong to the Caribbean/South America III clade and were probably imported from the Caribbean islands. The fourth lineage (BR-IV) belong to the Caribbean/South America I clade and was probably introduced from Colombia or Venezuela; while we found no evidence of dissemination into Brazil of the Caribbean/South America II lineage that mainly hits the Pacific side of the Andes (Peru, Ecuador and Colombia). Recent studies have shown the re-introduction into Brazil of a new DENV-2 lineage of the American/Asian genotype, that is closely related to DENV-2 strains circulating in some Caribbean islands (Martinique, Cuba and the Dominican Republic) [42], [43]. Thus, the Caribbean region seems to be the main source of new DENV strains introduced into Brazil.

The final outcome of each DENV-3 introduction into Brazil was highly variable. The most successful clade was the BR-I. This lineage comprises 86% of DENV-3 Brazilian sequences analyzed in the present study, was detected in all country regions from 2001 to 2009, and was also disseminated to other countries from the Southern Cone (Argentina, Bolivia and Paraguay). The lineage BR-II comprises 12% of Brazilian sequences and has been detected in the North region between 2003 and 2008, in Sao Paulo state (Southeast region) and Paraguay in 2006 and in Argentina in 2007. In contrast with the previous clades, the BR-III and BR-IV lineages seem to have failed to become established in Brazil, since both lineages were detected in one single individual each from the North region at 2002 and 2003, respectively, and none of the later Brazilian isolates grouped within these clades. The detection of DENV-3 lineages BR-I and BR-II in Argentina, Bolivia and Paraguay, points to Brazil as an efficient hub of dissemination of DENV-3 in the Southern cone.

Our phylogeographic analysis suggests that the most widely disseminated Brazilian DENV-3 clade (BR-I) probably entered into the country through the Southeast region. The state of Rio de Janeiro in the Southeast region is considered as the most important point for the introduction and dissemination of new DENV strains in Brazil as this sate was the place where the first cases of DENV-1 (1986), DENV-2 (1990), and DENV-3 (2000) were detected in Brazil [24]. The states of Rio de Janeiro and Sao Paulo also displayed among the largest and heavily dense urban population in the country, contain the most important national and international airports, and are highly connected to other states trough a large roadway and railway system. These conditions create an excellent milieu for introduction and rapid dissemination of new viral strains. On the other hand, lineages BR-II, BR-III, and BR-IV were probably introduced through the North Brazilian region. The close geographic proximity of the Northern states to the Caribbean region may explain the existence of a considerable diffusion of DENV-3 lineages across the northern Brazilian border.

The evolutionary analysis suggests that DENV-3 lineage BR-I displayed a period of cryptic circulation for about 1–3 years before its detection by the Brazilian public surveillance network. According to our estimates the BR-I clade emerged around 1999 (95% HPD: 1997–2000); but its presence was first reported in Rio de Janeiro at December 2000. In agreement with our findings, Romano *et al* [43] estimated that the new DENV-2 lineage detected in Rio de Janeiro at 2007 was introduced in the country at least 2–3 years earlier. Our analysis also supports that DENV-3 clades BR-I and BRII were disseminated at a very fast rate throughout Brazil. The reconstructed spatio-temporal pattern of DENV-3 dissemination indicates that lineages BR-I and BR-II emerged at around 1999 and 2001 in the Southeast and North regions, respectively; and only a few years later (2002–2006) both lineages were detected in regions located almost 5,000 km away. A similar finding was recently observed for DENV-4 that, after an absence of 30 years, was detected in Roraima state (North region) in July 2010 and only eight months later the virus was detected in several states of the Northeast and Southeast regions [44].

This study also points to the existence of a non-random pattern of DENV-3 dissemination across Brazilian regions, and further reveals significant differences in the molecular profile of DENV-3 epidemics occurring at the two most populated states of the country, Sao Paulo and Rio de Janeiro. The DENV-3 epidemics occurring in Sao Paulo state during the 2000s were seeded by the introduction and co-circulation of at least three viral strains (BR-SP-I, BR-SP-II, and BR-SP-III), consistent with previous observations [26], [27]. By contrast, the successive DENV-3 outbreaks taking place in Rio de Janeiro over the last decade were the result of the long-term persistence and *in situ* evolution of a single viral lineage (BR-RJ). Those DENV-3 strains detected in Sao Paulo were closely related to DENV-3 strains circulating in the Central-Western and Northern Brazilian regions. While, the BR-RJ lineage circulates in the states of Espirito Santo (Southeast region) and Pernambuco (Northeast region). Of note, despite the intense movement of people, high geographic proximity and dense viral sampling, we found no evidence of an important DENV-3 flux between Sao Paulo and Rio de Janeiro.

In conclusion, our study demonstrates that there have been at least four introductions of the same DENV-3 genotype III in Brazil, although only two viral lineages seems to have become efficiently established and disseminated in the country. The Caribbean islands were the main source of DENV-3 viruses that arrived into Brazil, and the Northern and Southeastern Brazilian regions seems to be most important hubs of introduction and dissemination of such DENV-3 lineages. Our analyses also suggest that DENV-3 strains circulated for at least 1–2 years until meet favorable conditions to initiate an outbreak and to be detected by the Brazilian public surveillance system. Continuous epidemiological surveillance and dense sequencing of viral strains circulating in all Brazilian regions are of paramount importance to early detection of newly emerging DENV lineages, to understanding the patterns of DENV dissemination across country regions, and to guide the actions for dengue control programs in Brazil.

## Supporting Information

Table S1 **DENV-3 sequences of Brazilian origin.**
(DOC)

## References

[1] Gubler DJ (2002) Epidemic dengue/dengue hemorrhagic fever as a public health, social and economic problem in the 21st century. Trends Microbiol 10: 100–103. 11827812 10.1016/s0966-842x(01)02288-0

[2] Gubler DJ (1998) Dengue and dengue hemorrhagic fever. Clin Microbiol Rev 11: 480–496. 9665979 10.1128/cmr.11.3.480 PMC88892

[3] Cologna R, Armstrong PM, Rico-Hesse R (2005) Selection for virulent dengue viruses occurs in humans and mosquitoes. J Virol 79: 853–859. 15613313 10.1128/JVI.79.2.853-859.2005 PMC538581

[4] Rico-Hesse R (2010) Dengue virus virulence and transmission determinants. Curr Top Microbiol Immunol 338: 45–55. 19802577 10.1007/978-3-642-02215-9_4 PMC3057078

[5] Vu TT, Holmes EC, Duong V, Nguyen TQ, Tran TH, et al (2010) Emergence of the Asian 1 genotype of dengue virus serotype 2 in viet nam: in vivo fitness advantage and lineage replacement in South-East Asia. PLoS Negl Trop Dis 4: e757. 20651932 10.1371/journal.pntd.0000757 PMC2907417

[6] Messer WB, Gubler DJ, Harris E, Sivananthan K, de Silva AM (2003) Emergence and global spread of a dengue serotype 3, subtype III virus. Emerg Infect Dis 9: 800–809. 12899133 10.3201/eid0907.030038 PMC3023445

[7] Dash PK, Parida MM, Saxena P, Abhyankar A, Singh CP, et al (2006) Reemergence of dengue virus type-3 (subtype-III) in India: implications for increased incidence of DHF & DSS. Virol J 3: 55. 16824209 10.1186/1743-422X-3-55 PMC1559593

[8] Kanakaratne N, Wahala WM, Messer WB, Tissera HA, Shahani A, et al (2009) Severe dengue epidemics in Sri Lanka, 2003–2006. Emerg Infect Dis 15: 192–199. 19193262 10.3201/eid1502.080926 PMC2662655

[9] Dorji T, Yoon IK, Holmes EC, Wangchuk S, Tobgay T, et al (2009) Diversity and origin of dengue virus serotypes 1, 2, and 3, Bhutan. Emerg Infect Dis 15: 1630–1632. 19861059 10.3201/eid1510.090123 PMC2866390

[10] Franco L, Di Caro A, Carletti F, Vapalahti O, Renaudat C, et al (2010) Recent expansion of dengue virus serotype 3 in West Africa. Euro Surveill 15. 20184854

[11] Usuku S, Castillo L, Sugimoto C, Noguchi Y, Yogo Y, et al (2001) Phylogenetic analysis of dengue-3 viruses prevalent in Guatemala during 1996–1998. Arch Virol 146: 1381–1390. 11556713 10.1007/s007050170098

[12] Miagostovich MP, dos Santos FB, de Simone TS, Costa EV, Filippis AM, et al (2002) Genetic characterization of dengue virus type 3 isolates in the State of Rio de Janeiro, 2001. Braz J Med Biol Res 35: 869–872. 12185377 10.1590/s0100-879x2002000800002

[13] Peyrefitte CN, Couissinier-Paris P, Mercier-Perennec V, Bessaud M, Martial J, et al (2003) Genetic characterization of newly reintroduced dengue virus type 3 in Martinique (French West Indies). J Clin Microbiol 41: 5195–5198. 14605161 10.1128/JCM.41.11.5195-5198.2003 PMC262480

[14] Peyrefitte CN, Pastorino BA, Bessaud M, Gravier P, Tock F, et al (2005) Dengue type 3 virus, Saint Martin, 2003–2004. Emerg Infect Dis 11: 757–761. 15890134 10.3201/eid1105.040959 PMC3320377

[15] Rodriguez-Roche R, Alvarez M, Holmes EC, Bernardo L, Kouri G, et al (2005) Dengue virus type 3, Cuba, 2000–2002. Emerg Infect Dis 11: 773–774. 15898173 10.3201/eid1105.040916 PMC3320353

[16] Aquino VH, Anatriello E, Goncalves PF, EV DAS, Vasconcelos PF, et al (2006) Molecular epidemiology of dengue type 3 virus in Brazil and Paraguay, 2002–2004. Am J Trop Med Hyg 75: 710–715. 17038699

[17] Kochel T, Aguilar P, Felices V, Comach G, Cruz C, et al (2008) Molecular epidemiology of dengue virus type 3 in Northern South America: 2000–2005. Infect Genet Evol 8: 682–688. 18674640 10.1016/j.meegid.2008.06.008

[18] Villabona-Arenas CJ, Miranda-Esquivel DR, Jimenez RE (2009) Phylogeny of dengue virus type 3 circulating in Colombia between 2001 and 2007. Trop Med Int Health 14: 1241–1250. 19619216 10.1111/j.1365-3156.2009.02339.x

[19] Araujo JM, Nogueira RM, Schatzmayr HG, Zanotto PM, Bello G (2009) Phylogeography and evolutionary history of dengue virus type 3. Infect Genet Evol 9: 716–725. 19010450 10.1016/j.meegid.2008.10.005

[20] CDC (1995) Dengue type 3 infection–Nicaragua and Panama, October-November 1994. MMWR Morb Mortal Wkly Rep 44: 21–24. 7808383

[21] Guzman MG, Vazquez S, Martinez E, Alvarez M, Rodriguez R, et al (1996) [Dengue in Nicaragua, 1994: reintroduction of serotype 3 in the Americas]. Bol Oficina Sanit Panam 121: 102–110. 8983243

[22] Ramirez A, Fajardo A, Moros Z, Gerder M, Caraballo G, et al (2010) Evolution of dengue virus type 3 genotype III in Venezuela: diversification, rates and population dynamics. Virol J 7: 329. 21087501 10.1186/1743-422X-7-329 PMC2998486

[23] Siqueira JB Jr, Martelli CM, Coelho GE, Simplicio AC, Hatch DL (2005) Dengue and dengue hemorrhagic fever, Brazil, 1981–2002. Emerg Infect Dis 11: 48–53. 15705322 10.3201/eid1101.031091 PMC3294356

[24] Nogueira RM, Miagostovich MP, de Filippis AM, Pereira MA, Schatzmayr HG (2001) Dengue virus type 3 in Rio de Janeiro, Brazil. Mem Inst Oswaldo Cruz 96: 925–926. 11685256 10.1590/s0074-02762001000700007

[25] Nogueira RM, Schatzmayr HG, de Filippis AM, dos Santos FB, da Cunha RV, et al (2005) Dengue virus type 3, Brazil, 2002. Emerg Infect Dis 11: 1376–1381. 16229765 10.3201/eid1109.041043 PMC3310608

[26] Amarilla AA, de Almeida FT, Jorge DM, Alfonso HL, de Castro-Jorge LA, et al (2009) Genetic diversity of the E protein of dengue type 3 virus. Virol J 6: 113. 19627608 10.1186/1743-422X-6-113 PMC2720943

[27] Mondini A, de Moraes Bronzoni RV, Nunes SH, Chiaravalloti Neto F, Massad E, et al (2009) Spatio-temporal tracking and phylodynamics of an urban dengue 3 outbreak in Sao Paulo, Brazil. PLoS Negl Trop Dis 3: e448. 19478848 10.1371/journal.pntd.0000448 PMC2682200

[28] Igarashi A (1978) Isolation of a Singh's Aedes albopictus cell clone sensitive to Dengue and Chikungunya viruses. J Gen Virol 40: 531–544. 690610 10.1099/0022-1317-40-3-531

[29] Gubler DJ, Kuno G, Sather GE, Velez M, Oliver A (1984) Mosquito cell cultures and specific monoclonal antibodies in surveillance for dengue viruses. Am J Trop Med Hyg 33: 158–165. 6364855 10.4269/ajtmh.1984.33.158

[30] Miagostovich MP, dos Santos FB, Fumian TM, Guimaraes FR, da Costa EV, et al (2006) Complete genetic characterization of a Brazilian dengue virus type 3 strain isolated from a fatal outcome. Mem Inst Oswaldo Cruz 101: 307–313. 10.1590/s0074-02762006000300015 16862328

[31] Thompson JD, Gibson TJ, Plewniak F, Jeanmougin F, Higgins DG (1997) The CLUSTAL_X windows interface: flexible strategies for multiple sequence alignment aided by quality analysis tools. Nucleic Acids Res 25: 4876–4882. 9396791 10.1093/nar/25.24.4876 PMC147148

[32] Posada D (2008) jModelTest: phylogenetic model averaging. Mol Biol Evol 25: 1253–1256. 18397919 10.1093/molbev/msn083

[33] Guindon S, Gascuel O (2003) A simple, fast, and accurate algorithm to estimate large phylogenies by maximum likelihood. Syst Biol 52: 696–704. 14530136 10.1080/10635150390235520

[34] Guindon S, Lethiec F, Duroux P, Gascuel O (2005) PHYML Online–a web server for fast maximum likelihood-based phylogenetic inference. Nucleic Acids Res 33: W557–559. 15980534 10.1093/nar/gki352 PMC1160113

[35] Ronquist F, Huelsenbeck JP (2003) MrBayes 3: Bayesian phylogenetic inference under mixed models. Bioinformatics 19: 1572–1574. 12912839 10.1093/bioinformatics/btg180

[36] Rambaut A, Drummond A (2007) Tracer v1.4. Available from http://beastbioedacuk/Tracer.

[37] Drummond AJ, Nicholls GK, Rodrigo AG, Solomon W (2002) Estimating mutation parameters, population history and genealogy simultaneously from temporally spaced sequence data. Genetics 161: 1307–1320. 12136032 10.1093/genetics/161.3.1307 PMC1462188

[38] Drummond AJ, Rambaut A (2007) BEAST: Bayesian evolutionary analysis by sampling trees. BMC Evol Biol 7: 214. 17996036 10.1186/1471-2148-7-214 PMC2247476

[39] Lemey P, Rambaut A, Drummond AJ, Suchard MA (2009) Bayesian phylogeography finds its roots. PLoS Comput Biol 5: e1000520. 19779555 10.1371/journal.pcbi.1000520 PMC2740835

[40] Drummond AJ, Rambaut A, Shapiro B, Pybus OG (2005) Bayesian coalescent inference of past population dynamics from molecular sequences. Mol Biol Evol 22: 1185–1192. 15703244 10.1093/molbev/msi103

[41] Drummond AJ, Ho SY, Phillips MJ, Rambaut A (2006) Relaxed phylogenetics and dating with confidence. PLoS Biol 4: e88. 16683862 10.1371/journal.pbio.0040088 PMC1395354

[42] Oliveira MF, Galvao Araujo JM, Ferreira OC Jr, Ferreira DF, Lima DB, et al (2010) Two lineages of dengue virus type 2, Brazil. Emerg Infect Dis 16: 576–578. 20202456 10.3201/eid1603.090996 PMC3322019

[43] Romano CM, de Matos AM, Araujo ES, Villas-Boas LS, da Silva WC, et al (2010) Characterization of Dengue virus type 2: new insights on the 2010 Brazilian epidemic. PLoS One 5: e11811. 20676363 10.1371/journal.pone.0011811 PMC2911371

[44] Nogueira RM, Eppinghaus AL (2011) Dengue virus type 4 arrives in the state of Rio de Janeiro: a challenge for epidemiological surveillance and control. Mem Inst Oswaldo Cruz 106: 255–256. 21655810 10.1590/s0074-02762011000300001

