# Supplementary material for: Origin and Evolution of Dengue Virus Type 3 in Brazil
Source: PLoS Negl Trop Dis. 2012 Sep 6;6(9):e1784. doi: 10.1371/journal.pntd.0001784 (PMC3435237; doi:10.1371/journal.pntd.0001784)
Supplement: Table S1 — DENV-3 sequences of Brazilian origin. (DOC) [file pntd.0001784.s001.doc]

| **Region** | **State** | ***N*** | **Sampling dates** |
| --- | --- | --- | --- |
| Southeast | Sao Paulo | 58 | 2003-2009 |
|  | Rio de Janeiro | 13 | 2001-2008 |
|  | Espirito Santo | 3 | 2003-2005 |
| North | Rondonia | 6 | 2002-2003 |
|  | Para | 5 | 2003-2004 |
|  | Amazonas | 1 | 2002 |
|  | Roraima | 1 | 2002 |
|  | Not specified | 7 | 2003-2008 |
| Central-West | Goias | 6 | 2002-2006 |
|  | Mato Grosso | 1 | 2002 |
|  | Not specified | 1 | 2001 |
| Northeast | Pernambuco | 4 | 2002-2003 |
|  | Maranhão | 1 | 2002 |
